# Nanoformulations with exopolysaccharides from cyanobacteria: enhancing the efficacy of bioactive molecules in the Mediterranean fruit fly control

**DOI:** 10.1007/s11356-023-28180-x

**Published:** 2023-06-22

**Authors:** Sara Falsini, Marzia Cristiana Rosi, Elia Ravegnini, Silvia Schiff, Cristina Gonnelli, Alessio Papini, Alessandra Adessi, Silvia Urciuoli, Sandra Ristori

**Affiliations:** 1 Dipartimento di Biologia, Università degli studi di Firenze, via P.A. Micheli 1-3, 50121 Firenze, Italy; 2 Dipartimento di Scienze e Tecnologie Agrarie, Alimentari, Ambientali e Forestali, DAGRI, Università degli Studi di Firenze, Via Maragliano 77, 50144 Firenze, Italy; 3 Laboratorio PHYTOLAB (Pharmaceutical, Cosmetic, Food supplement Technology and Analysis), DiSIA, Dipartimento di Statistica, Informatica, Applicazioni “Giuseppe Parenti”, Università degli Studi di Firenze, Polo Scientifico e Tecnologico via U. Schiff, 6, 50019 Sesto Fiorentino, FI Italy; 4 Dipartimento di Chimica “Ugo Schiff” and CSGI, Università di Firenze, Via della Lastruccia 3, 50019 Sesto Fiorentino, Firenze Italy

**Keywords:** Dynamic light scattering, Zeta Potential, *Ceratitis capitata*, Nanopesticides, Bioactive molecules, Capsaicin, Hydroxytyrosol

## Abstract

**Abstract:**

The increasing demand for food has required intensive use of pesticides which are hazardous to the ecosystem. A valid alternative is represented by biopesticides; however, these molecules are often insoluble in water, and poorly bioavailable. Nanopesticides can be engineered to reach a selected target with controlled release of the active principle. In this work, capsaicin, an irritant alkaloid from hot chili peppers, and hydroxytyrosol, a phenolic compound obtained from extra-virgin olive oil by-products, were loaded into innovative nanocarriers. These were designed ad hoc combining exopolysaccharides from the cyanobacteria *Neocyanospira capsulata*, and a lipid component, i.e., egg phosphatidylcholine. The polysaccharide was chosen for chemical affinity with the chitin of insect exoskeleton, while the lipids were introduced to modulate the carrier rigidity. The newly formed nanosystems were characterized by physico-chemical techniques and tested for their possible use in pest control programs. The Mediterranean Fruit Fly *Ceratitis capitata* Wiedemann, 1824 (Diptera, Tephriditae), a pest of the Mediterranean Region causing high economic losses, was used as a model insect. We found that the nanoformulations nanocarriers prepared in this work, were able to increase the ovicidal effect of hydroxytyrosol. Moreover, the formulation encapsulating either hydroxytyrosol or capsaicin were able to reduce the number of females landing on treated apricots.

**Graphical Abstract:**

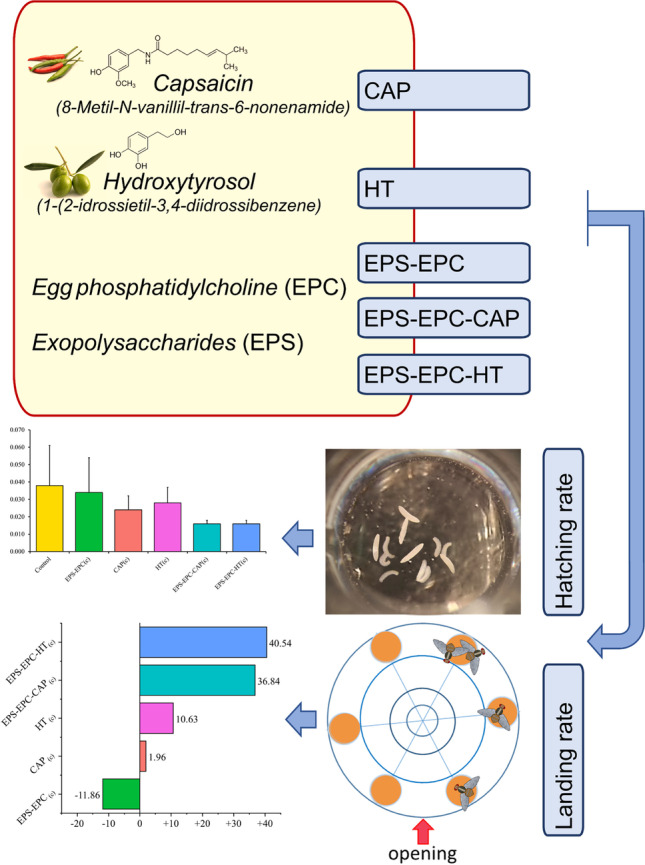

**Supplementary Information:**

The online version contains supplementary material available at 10.1007/s11356-023-28180-x.

## Introduction

Pesticides are widely used in agriculture and have become an essential factor to reduce the loss of crops due to pests and pathogens (Food and Agriculture Organization of the United Nations (FAO) 2007; Zhao et al. 2018). The appropriate use of pesticides can preserve the yield, restoring up to 30% of the total harvest all over the world, according to the Food and Agriculture Organization of the United Nations (FAO) data (Lamberth et al. 2013). On the other hand, to meet the increasing demand for continuous population growth, the intensive use of pesticides inevitably pollutes the environment, affecting the abiotic and biotic components (Fang et al. 2023).

Currently, research has led to the discovery of new bioactive compounds, i.e., secondary metabolites produced by plants as deterrents and control agents for pests (Zaynab et al. 2018), with the advantages of being biodegradable and less harmful compared to synthetic pesticides (Rajput et al. 2021). Unfortunately, the majority of these molecules are water-insoluble, photolabile, and thus degradable if not properly vehiculated (Lowry et al. 2019). In this context, nanotechnology offers a range of possibilities for properly designed engineered nanoformulations to guarantee the release of the natural compounds to the target, also reducing their dispersion in the environment. Recently, biopolymers have gained attention as carriers for their biocompatibility, versatility, sustainable release of natural compounds, and their low impact on the environment (Kumar et al. 2022). Several biopolymers can be used for phytochemicals delivery, such as lignin (Falsini et al. 2019), tannins (Falsini et al. 2023), starch, alginate, and exopolysaccharides (EPS). These latter derive from the polysaccharide material released into the culture medium during cell growth of specific cyanobacteria strains (Vincenzini et al. 1990)*.* Because of their biochemical properties and stable production over time, these natural polymers show several advantages in view of possible industrial applications (De Philippis and Vincenzini 1998; Pereira et al. 2009), especially considering the need for new sustainable approaches (Vincenzini et al. 1990). Among different species, *Neocyanospira capsulata* (Florenzano, Sili, Pelosi and Vincenzini) Molinari and Guiry (formerly *Cyanospira capsulata*), a heterocystous, akinete-forming cyanobacterium isolated from the alkaline soda Lake Magadi (Kenya) (Florenzano et al. 1985), is one of the most studied. *N. capsulata* releases an acidic exopolysaccharide with two different uronic acid groups that are responsible for the disclosure of any ordered structures after inducing variation of pH and/or ionic strength (Cesàro et al. 1990).

Regarding bioactive compounds extracted from plants, flavonoids, polyphenols, and alkaloids are widely used as biopesticides (Lamberth et al. 2013). For example, capsaicin (CAP) is an alkaloid that confers a spicy flavour to chili peppers. This molecule exerts toxicity and repellence on spider mites (Antonious et al. 2006) and works as an oviposition deterrent against other insects, such as *Delia antiqua* (onion fly) (Cowles et al. 1898), *Callosobruchus maculatus* (Lale 1992), and *Drosophila melanogaster* (Li et al. 2020)*.* Moreover, acting as irritant, capsaicin influences the social behaviour of insects and, in particular, inhibits the foraging of beetles, like *Tenebrio molitor* (mealworm) (Olszewska and Tęgowska 2011). The precise mechanisms underlying capsaicin's effects on insects remain partially known, likely involving multiple mechanisms such as altered heat nociception (Maliszewska et al. 2018; Olszewska and Tęgowska 2011) or disruption of hemolymph buffering (Maliszewska et al. 2019). In the case of D. suzukii, capsaicin induces a potent aversive response in female insects by activating nociceptive neurons. Furthermore, capsaicin can potentially impact the lifespan of flies by causing intestinal dysplasia and activating genes linked to innate oxidative immunity (Li et al. 2020).

Among the phenolic compounds of *Olea europaea* L. present in extra-virgin olive oil and its by-products, the phenolic alcohol hydroxytyrosol (HT) has been discovered to have beneficial effects as an antioxidant, stabilizing reactive oxygen species (ROS), specifically oxygen radicals with intramolecular hydrogen bound (Sroka and Cisowski 2003). Recently the application of HT has been extended an inhibitor of bacteria (Biancalani et al. 2016), fungi (Drais et al. 2021) and plant pests insects. In particular, hydroxytyrosol-rich preparations and other polyphenolic compounds derived from olive mill wastewater exhibit insecticidal, repellent, and antifeedant properties against various insect pests (Debo et al. 2011). These compounds demonstrate ovicidal effects, impair the fecundity of female insects, and disrupt their growth and development due to histopathological effects on the alimentary canal (Di Ilio and Cristofaro 2021). Moreover, hydroxytyrosol-rich preparations inhibit digestive enzymes, alter hemolymph metabolite levels, and influence enzyme activity (Abdellaoui et al. 2019). In this work, innovative nanoformulations were designed to carry the two active molecules described above, i.e., capsaicin and hydroxytyrosol, mixing exopolysaccharides from *Neocynanospira capsulata* and purified lipids. The former component has an affinity with the saccharides present in the insect's exoskeleton, while, the latter can be used to control the nanocarrier morphology and stability, as already shown in previous works on formulations obtained from the olive pomace (Clemente et al. 2018; Clemente et al. 2019). In addition, lipids contribute to reducing the highly hydrophilic nature of saccharide units, providing a more hydrophobic environment in the nanocarrier, where the active molecules can be loaded.

The target organism chosen is *Ceratitis capitata* Wiedemann, 1824 (Medfly), which is spread worldwide and, in particular, in the Mediterranean area, where it attacks more than 250 varieties of fruits (Fimiani 1989). Depending on the environmental conditions, the *C. capitata* life cycle lasts 20–90 days (Metcalf and Flint 1962). After mating, females lay 2–20 eggs under the fruit’s epidermis, where larvae develop feeding on the pulp, thus causing the loss of yield. *C. capitata* control program has been mostly based on the large-scale use of conventional insecticides, most of which are currently under banning (Di Ilio and Cristofaro 2021). In the control of medflies, pyrethroids have become widely used insecticides. However, the extensive use of chemical insecticides has raised concerns about their impact on the environment, toxicology, and the development of resistance (Castells-Sierra et al. 2023). This emphasizes the necessity for alternative control strategies that minimize adverse effects on non-target organisms and the environment. This aspect opened the way to test natural compounds as an alternative strategy for medfly control (Benelli et al. 2012).

Accordingly, our aims were (i) designing ad hoc nanovectors transporting capsaicin or hydroxytyrosol obtained by the mixing of eco-friendly components such as exopolysaccharides with phosphatidylcholine as helper lipids; (ii) performing in-depth physico-chemical characterization of the newly obtained nanosystems; (iii) testing the formulations on *C. capitata* used as a model insect, to investigate their toxicity on eggs and the first larvae instar, and their influence on the landing deterrence on host fruit.

## Materials and methods

### Experimental materials

The exopolysaccharides (EPS) (2 MDa) were obtained by the blue-green cynobacteria *Neocyanospira capsulata,* a strain of which was present in the culture collection of the Department of Agriculture, Food, Environment and Forestry (Microbiology Section) of the University of Florence, following the methodology described by Vincenzini et al. (1990). Egg phosphatidylcholine (EPC) was purchased from Avanti Polar Lipids Inc., Alabaster, AL. Capsaicin (CAP), with Molecular Weight (MW) of 305.41 g/mol, was purchased by Merck (Darmstadt, Germany) as well as all the solvents used. Hydroxytyrosol (HT), with MW of 154.16 g/mol, was extracted from the free-oil olive pulp at the PHYTOLAB, University of Florence, following the procedure described by Romani et al. (2017).

### Nanosystems preparation

Nanosystems were prepared in round bottom vials by mixing 600 μL of EPC stock solution, which was 3.4 × 10^−2^ M in chloroform, with a volume of 1.5 mL of CAP or HT solutions, whose concentrations were 3.3 × 10^−2^ M in acetone. Dry lipid films (EPC), with or without bioactive molecules, were obtained by evaporating the solvent under vacuum overnight. Then, they were rehydrated with 3 mL of EPS solution at a concentration of 2.5 mg/mL or 0.5 mg/mL. All the obtained samples were subjected to a first homogenization by five freeze-thaw cycles in liquid nitrogen and water bath at 50 °C and then sonication by 5 cycles of 3 min (75% power level with a Bandelin Electronic Sonopuls, Berlin, Germany). In concentrated samples, the EPC and EPS content was 2 × 10^−2^ M and 2.5 mg/mL, respectively. In loaded nanosystems, the concentration of CAP and HT was 1.6 × 10^−2^M.

The acronyms of the samples investigated in this study are listed in Table 1.

**Table 1:** Samples acronym and composition

	Sample	EPS concentration (mg/mL)	EPC concentration (M)	CAP concentration (M)	HT concentration (M)
Empty vector	EPS-EPC_(d)_	0.5	1 10^−3^	–	–
	EPS-EPC_(c)_	2.5	2 10^−2^	–	–
Loaded vector	EPS-EPC-CAP_(d)_	0.5	1 10^−3^	3.2 10^−4^	–
	EPS-EPC-CAP_(c)_	2.5	2 10^−2^	1.6 10^−2^	
	EPS-EPC-HT_(d)_	0.5	1 10^−3^		3.2 10^−4^
	EPS-EPC-HT_(c)_	2.5	2 10^-2^	–	1.6 10^−2^

*EPS* exopolysaccharides, *EPC* egg phosphatidylcholine, *HT* hydroxytyrosol, *CAP* capsaicin, *d* diluted, *c* concentrated, *ns* not sonicated

### Nanosystems characterization

#### Dynamic light scattering (DLS)

Dynamic light scattering measurements were performed on a Malvern Zetasizer Nano ZS (ZEN 1600 model, Malvern Instruments Southborough, MA), equipped with He-Ne 633, 4 mW laser with backscattering detection. DLS experiments were performed over 11 runs and in duplicate. Samples were diluted at 1:200 with Milli-Q water before measuring to adjust turbidity.

#### Zeta potential (ζ)

Zeta potential (ζ) measurements were performed on a Zatasizer (Zetasizer Pro, Malvern Panalytical Co. Ltd., Malvern, UK) in DTS1070 cells, at 25 °C. The **ζ** values were measured in a mixed mode, using phase analysis light scattering (M3-PALS). Samples were diluted at 1:100 with Milli-Q water. The measurement was repeated 3 times, and values are presented as the mean ± SD.

### Insects rearing

The population of *C. capitata* was reared in the laboratory of the Department of Agriculture, Food, Environment and Forestry of the University of Florence (as in Granchietti et al. 2012). Adult insects were maintained inside net cages with dimensions 30 × 30 × 30 cm, located in a chamber with controlled conditions, i.e., 14 h photoperiod, a temperature of 25 ± 1 °C, and RH of 60 ± 10%. Adult insects were fed with water and a solid diet consisting of sucrose, yeast, and enzymatic yeast hydrolysate in a 6:2:1 ratio (Cavalloro and Girolami 1969). The adult females lay the eggs passing the ovipositor through the net cage. Eggs fall into a collection container with tap water placed under the cage (Economopoulos and Judt 1989).

### Insect assays

#### Eggs and first-stage larval toxicity

Eggs freshly laid within 1 hour were gathered from a collection vessel placed under the rearing cage and then randomly selected to be individually transferred in the pits of a 96-well plate, following the protocol of Di Ilio and Cristofaro (2021) with minor adjustments. In particular, ten eggs were located in each well filled with 80 μL of formulations, as listed in Table 2; two sets of experiments were realized at different concentrations: EXP_(d)_ and EXP_(c)_. Distilled water was used for the control group, and twelve replicates were performed for each treatment. Experiments lasted 5 days, during which newly hatched first larval instars were counted day-to-day. The hatching rate was determined as the ratio between the first instar larvae obtained after 72 h and the initial number of eggs. We further investigated the effects of nanosystems on the first instar larvae; during the trial, the first instar dead larvae were counted to determine the survival rate. Insects were examined through a stereomicroscope equipped with a fibre-optic illuminator (Leica/Wild M3Z equipped with L2 illuminator; Leica Microsystems, Wetzlar, Germany) to verify whether they were alive; larvae with a shrivelled body and chromatic variation were considered dead.

**Table 2:** Experimental layout of natural molecules and nanoformulations used in the eggs toxicity assays with diluted (d) and concentrated (c) bioactive molecules

Experimental name	Tested sample	EPS (mg/mL)	EPC (M)	Bioactive molecules (M)
EXP_(d)_	CAP_(d)_	–	–	3.2 10^−4^
	HT_(d)_	–	–	3.2 10^−4^
	EPS-EPC_(d)_	0.5	1 10^-3^	
	EPS-EPC-CAP_(d)_	0.5	1 10^-3^	3.2 10^−4^
	EPS-EPC-HT_(d)_	0.5	1 10^-3^	3.2 10^−4^
EXP_(c)_	CAP_(c)_	–	–	1.6 10^−2^
	HT_(c)_	–	–	1.6 10^−2^
	EPS-EPC_(c)_	2.5	2 10^-2^	–
	EPS-EPC-CAP_(c)_	2.5	2 10^-2^	1.6 10^−2^
	EPS-EPC-HT_(c)_	2.5	2 10^-2^	1.6 10^−2^

#### Landing preference of *C. capitata* females on apricots

**Fig. 1:**
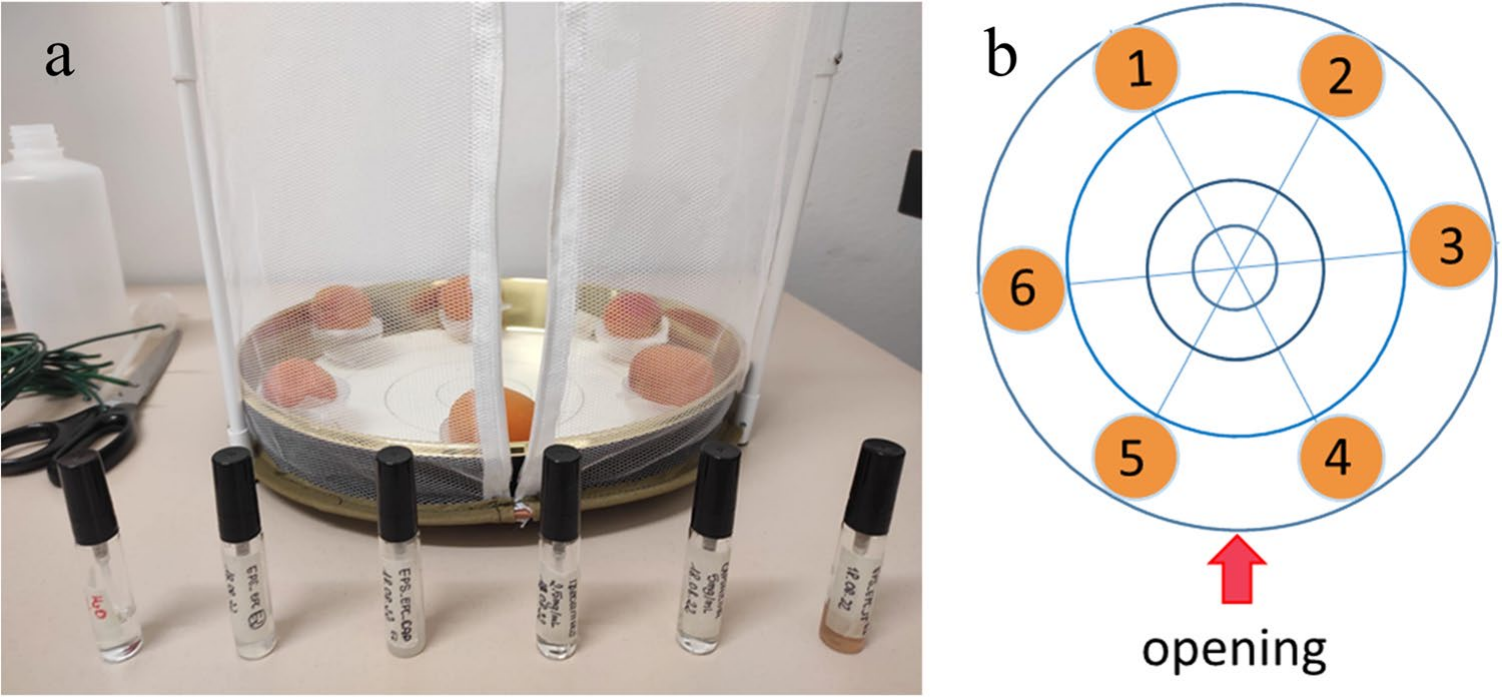
**a** Cage with apricots sprayed with each different treatment and **b** randomized disposition of the apricots considering the opening as a reference point

The landing preference was assayed in a multiple-choice test on organic apricots with trademark COOP ViviVerde Bio. In this experiment, bioassays were conducted using natural molecules and nanoformulations at their highest concentrations. Each fruit was positioned on a plastic support and sprayed with treatments indicated in Table 2 for EXP_(c)_, including distilled water, as control. Fruits, visually selected for homogeneous shape, dimension, and colour, were placed at the bottom of a cylindrical screen cage (30 cm of diameter and height of 40 cm). Thus, seventeen females (5/10-day-old) of *C. capitata*, randomly selected from the rearing, were placed into the cage where apricots were randomly disposed in each cage to control position effects and possible differences among fruits (Fig. 1B). Medflies were left undisturbed for 45 min to allow acclimatization; then, females were observed every 30 min, taking a picture of the insect position either on treated or untreated fruits. The females’ choice was recorded when females lasted on the same fruit for 30 min or longer, and the landing rate was determined as the ratio between the number of females observed on the apricot and the total number of females present in the cages. The bioassay was stopped after 240 min, and the experiment was done in 6 replicates.

### Statistical analysis

Statistical analyses were done within R software version 3.5.1 (R Core Team 2022). The hatching rate for each egg toxicity investigation did not follow a normal distribution and respect the equality of variances; therefore, data were evaluated using a rank-based nonparametric multiple contrast test procedure (MCTP) and computation of compatible Simultaneous Confidence Intervals SCI (95%) (Noguchi et al. 2012; Noguchi et al. 2020) using the “nparcomp” package (Konietschke et al. 2015). This approach imposes no restrictive distributional assumptions by controlling the familywise error rate and using simultaneous confidence intervals (SCI) plus multiplicity-adjusted *p* values to make accurate comparisons. The comparison was adjusted using Tukey (every sample between them) contrast method and Fisher approximation. Simultaneous all-pairs comparisons are made for *p* = 0.05 level.

The nonparametric Kaplan-Meier method was applied to estimate the *C. capitata* first instar larval survival probability over time when exposed to the treatments verifying the null hypothesis of no difference between survival curves at any time point (*P* value > 0.05). The log-rank test (LRT) was applied to perform pairwise comparisons. Data were analyzed using the “survival” (version 3.5.0) and “survminer” (version 0.4.9) packages (Kassambara et al. 2022; Therneau 2023).

The female landing rate was evaluated using the rank-based MCT procedure, and simultaneous confidence intervals (95%) previously described. The effect of the treatments on deterring the landing of the fruits by *C. capitata* females was evaluated by calculating a deterrence index (DI) according to the following formula: DI = 100 × (control − treatement)/(control + treatement) (control = total females on control fruits; treatment = total females on treated fruits). The positive value of the index indicates that more females visited the control fruit than treated ones; a negative value is obtained when more females visited the treated fruit, and zero means that an equal number of females visited the treated and control fruits (Huang 1994).

## Results

### Dynamic light scattering (DLS) and zeta potential

The average diameter and polydispersity index of the mixed EPS-EPC nanoformulations prepared in this work are reported in Table 3. From DLS measurements, it can be evidenced that combining EPC lipids with EPS allowed the formation of nanostructures with smaller sizes. Indeed, mixed aggregates had a mean diameter of 150-200 nm, while particles formed by pure polysaccharide almost reached a micron. These differences indicated that only the lipid-containing particles are suited for drug delivery. Moreover, nanocarriers loaded with CAP or HT showed a slightly larger size in comparison to the empty nanovectors, suggesting that bioactive molecules could bring a small change in the nanoformulations morphology.

**Table 3:** Average diameter and polydispersity index (PDI) of the nanoparticles and zeta potential (**ζ)** (mV) of the nanoparticles

	Tested samples	Average diameter, nm	PDI	ζ, mV
Only EPS	EPS_(c)_	940 ± 50	0.89	−29 ± 2
Empty vector	EPS-EPC_(c)_	146 ± 5	0.31	−28 ± 0.8
Loaded vector	EPS-EPC-CAP_(c)_	153 ± 10	0.40	−26 ± 1.8
	EPS-EPC-HT_(c)_	192 ± 5	0.34	−16 ± 1.2

The surface of all the samples investigated in this work was negatively charged, as indicated by the **ζ** values reported in Table 3. In particular, EPS-EPC nanovectors were around – 30 mV, that is similar to the capsaicin-loaded nanoparticles, while the EPS-EPC-HT aggregates showed a surface charge of – 16 mV, indicating a different molecular packing induced by HT at the interface with the water medium. In all cases, the zeta potential values were indicative of stable formulations, which was in agreement with the observed behaviour.

### Eggs and first larval toxicity

The hatching rates of the eggs or larvae used in the control experiments were 0.85 and 0.89 in the EXP_(d)_ and EXP_(c)_, respectively, and they were comparable to the value reported by Rössler (1975) for wild and laboratory strains.

**Fig. 2:**
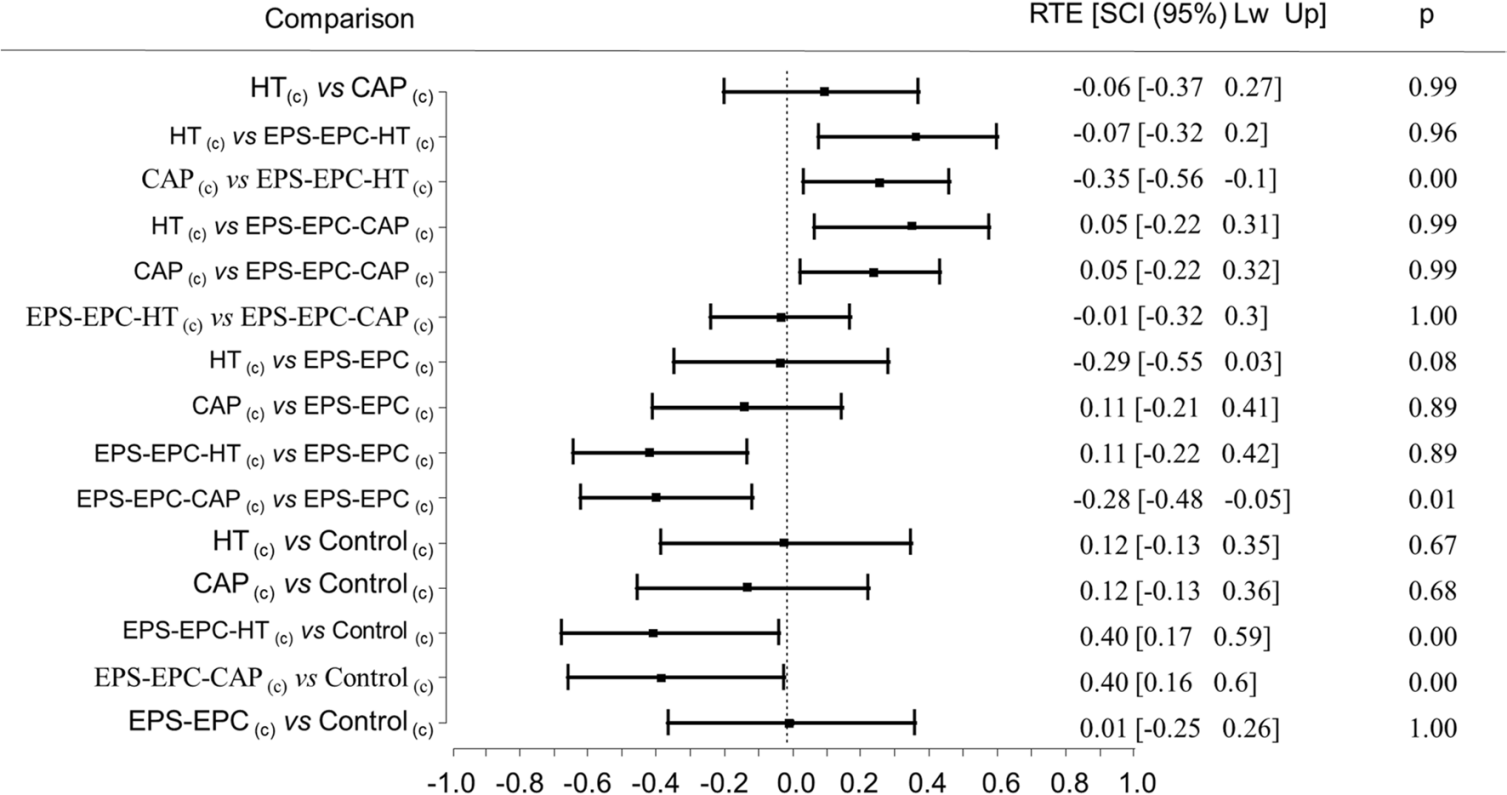
Forest plot and results of the nonparametric MCTP analyses for the specific contrasts of the natural bioactive molecules and nanoformulations (c) concentrated treatments on *C. capitata* eggs hatching rate. RTE = relative treatment effects size; SCI = effect size simultaneous confidence intervals; p = adjusted p-value. Comparison adjusted using Tukey contrast method and Fisher approximation

However, in the EXP_(d)_, all samples did not induce a significant reduction (*Q* = 3.06, *p* = 0.53) in larval hatching (Table 4). On the contrary, in the EXP_(c)_, MCT procedure and CI (95%) simultaneous comparison showed a highly significant difference for overall comparison (*Q* = 3.09; *p* < 0.001) and for pairwise contrasts (results reported in Table 4 and Fig. 2). The EPS-EPC-HT_(c)_ had superior ovicidal properties on medfly eggs compared to all other treatments and, specifically, showed an improved activity compared to hydroxytyrosol HT_(c)_ (mean = 0.91 ± 0.11). The results of the two experiments allowed us to theorize that the formulation efficacy conveyed in the EPS-EPC system could act in a dose-dependent ratio.

**Table 4:** Average hatching rate of *C. capitata* eggs expressed as a percentage and the corresponding variation coefficient (CV), along with the estimated effects per group and results for the overall comparisons

Experiments	Treatments	Hatching rate (%)	CV (%)	Effect size	Lower	Upper	*Q*	*p*
EXP_(d)_	Control	85.00	11.76	0.50	0.40	0.60	3.06	0.53
	EPS-EPC_(d)_	84.38	14.29	0.50	0.40	0.60		
	CAP_(d)_	78.98	18.99	0.39	0.29	0.51		
	HT_(d)_	86.42	12.79	0.54	0.43	0.64		
	EPS-EPC-CAP_(d)_	88.13	13.64	0.58	0.47	0.69		
	EPS-EPC-HT_(d)_	84.00	16.67	0.49	0.38	0.61		
EXP_(c)_	Control	89.43	11.24	0.56	0.46	0.66	3.09	0.00
	EPS-EPC_(c)_	84.92	18.82	0.50	0.38	0.62		
	CAP_(c)_	91.88	8.70	0.61	0.52	0.70		
	HT_(c)_	91.25	12.09	0.61	0.52	0.70		
	EPS-EPC-CAP_(c)_	86.88	11.49	0.49	0.41	0.58		
	EPS-EPC-HT_(c)_	66.67	28.36	0.21	0.15	0.29		

Number of samples per treatment *n* = 16, *Effect size* = effect size per group, *Lower* = lower bounds of the effect size, *Upper* = upper bounds of the Effect size, *Q* = quantiles of the adjusted *p* values for the overall comparisons, *p* = significance level of the overall comparisons

**Fig. 3:**
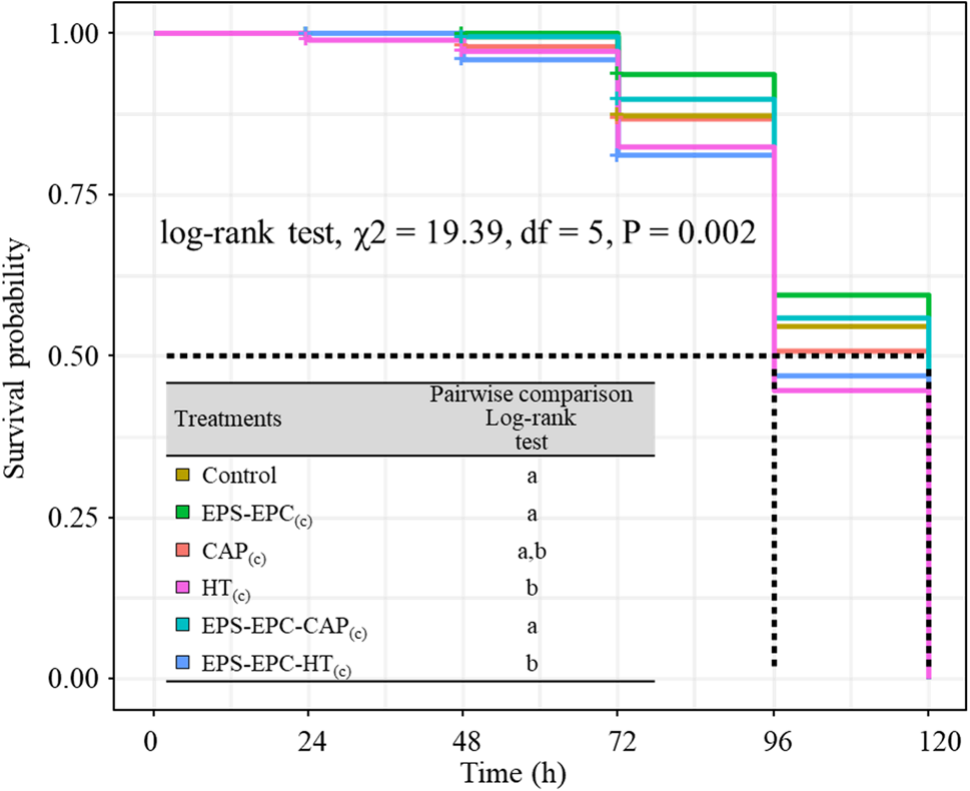
Kaplan–Meier survival curves for *C. capitata* first instar larvae exposed to distilled water (control), exopolysaccharides, phosphatidylcholine and bioactive molecules CAP_(c)_, HT_(c)_, and their nano formulation EPS-EPC-CAP_(c)_ and EPS-EPC-HT_(c)_. The horizontal dashed line at a survival probability of 0.5 represents the estimated median survival time (MST). The dashed vertical lines represent the median survival time (MST) expressed in hours (h) for the first instar larvae in each treatment group (control = 120; EPS-EPC_(c)_ = 120; HT_(c)_ = 96; EPS-EPC-CAP_(c)_ = 120; EPS-EPC-HT_(c)_ = 96). In the table, the results of the pairwise comparisons of survival curves performed by the log-rank test are presented. Different letters show significant differences in the log-rank test

In evaluating the first-age larvae survival, the experiment with molecules at the lower concentrations (3.2 10^−4^) did not show differences in the larval survival probability curves of the treatments (log-rank test, *χ*2 = 9.3, df = 5, *P* = 0.1), that was instead evident in EXP_(c)_, with natural molecules and nanosystems at higher concentrations (log-rank test, *χ*2 = 19.39, df = 5, *P* = 0.002). Higher larval mortality was evidenced in HT_(c)_, CAP_(c)_, and EPS-EPC-HT_(c)_ treatments (log-rank test pairwise comparison) (Fig. 3). Hydroxytyrosol seemed less favorable to larval survival; in fact, both HT_(c)_ and EPS-EPC-HT_(c)_ induced higher mortality starting from 72 h, and the 50% probability of survival occurred at 96 h, while in the other treatments was reached after 120 h. However, the effect of the EPS-EPC-HT_(c)_ loaded vector did not seem to be notably different compared to the natural molecule (Fig. 3).

### Landing preference of *C. capitata* females on apricots

**Fig. 4:**
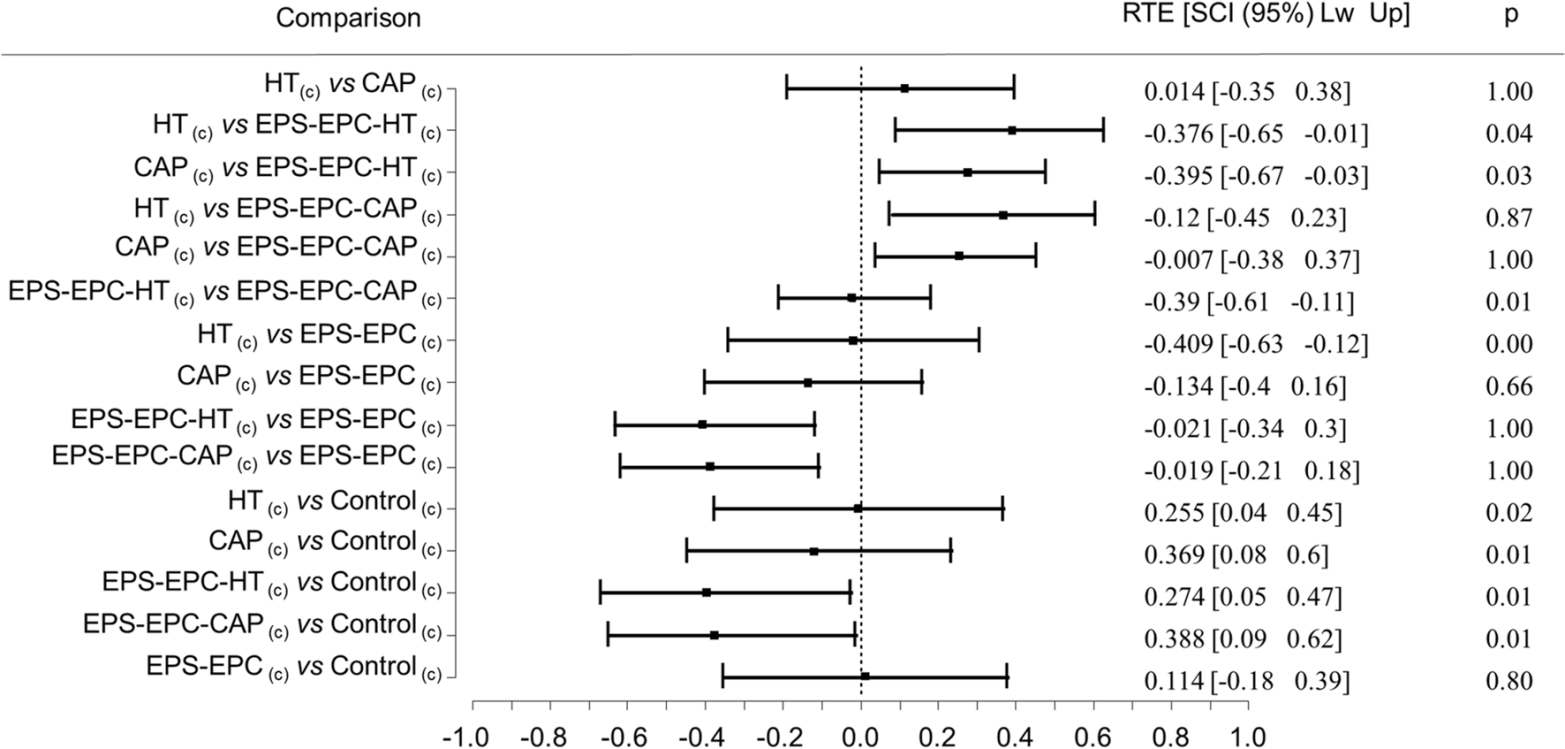
Forest plot and results of the nonparametric MCTP analyses for the specific contrasts of the natural bioactive molecules and nanoformulations (c) concentrated treatments on *C. capitata* female landing rate. RTE = relative treatment effects size; SCI = effect size simultaneous confidence intervals; *p* = adjusted *p* value. Comparison adjusted using Tukey contrast method and Fisher approximation

The landing rate of *C. capitata* females on apricots tended to be lower in nanoformulations than in other treatments with the same average value (0.016 ± 0.002) as indicated in Table 5. Compared to the control and the vector, the number of females that arrived on the fruit was almost the half (average value 0.038 ± 0.023 for the control and average value 0.034 ± 0.020 for the vector EPS-EPC_(c)_). Significant differences were, therefore, found in all comparisons considered; in particular, the two nanovectors used were more effective than the natural bioactive molecules CAP_(c)_ and HT_(c)_ (results shown in Fig. 4).

**Table 5:** Means and standard deviation of *C. capitata* female landing rate with the MTC estimated effects per group and results for the overall comparisons

Treatments	*n*	Mean±sd	Size effect	Lower	Upper	*Q*	*p*
Control	12	0.038±0.023	0.661	0.536	0.766	2.947	0.000
EPS-EPC_(c)_	16	0.034±0.020	0.643	0.545	0.731		
CAP_(c)_	17	0.024±0.008	0.504	0.420	0.588		
HT_(c)_	12	0.028±0.009	0.607	0.504	0.702		
EPS-EPC-CAP_(c)_	12	0.016±0.002	0.292	0.257	0.330		
EPS-EPC-HT_(c)_	11	0.016±0.002	0.292	0.257	0.330		

Number of samples per treatment: Control *n* = 12, EPS-EPC(c) *n* = 16, CAP(c) *n* = 17, HT(c) *n* = 12, EPS-EPC-CAP(c) *n* = 12, EPS-EPC-HT(c) *n* = 11, *Effect size* = effect size per group, *Lower* = lower bounds of the effect size, *Upper* = upper bounds of the effect size, *Q* = quantiles of the adjusted p-values for the overall test, *p* = significance level of the overall test. Comparison adjusted using Tukey contrast method and Fisher approximation

**Fig. 5:**
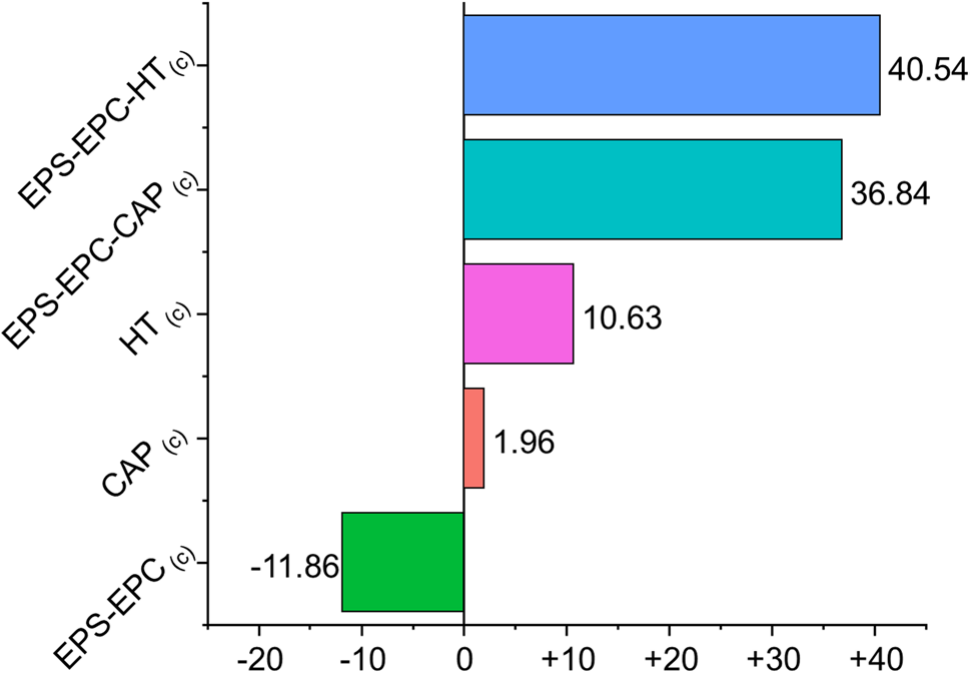
Results of the deterrence index (DI) of the natural bioactive molecules and the nanoformulation (c) concentrated effect on the females of *C. capitata* settlement on treated fruits

The deterrence index (DI) showed a robust landing deterrence of both nanovectors_(c)_ EPS-EPC-CAP_(c)_ (DI = 40.00) and EPS-EPC-HT_(c)_ (DI = 43.59) higher than the effect obtained with natural molecules CAP_(c)_ (DI = 5.66) and HT_(c)_ (DI = 14.29). A small attractive effect (DI = − 8.20) can be noted for the vector EPS-EPC_(c)_ (Fig. 5). This observation means that both EPS-EPC-CAP(c) and EPS-EPC-HT(c) led to lower females landing on the apricot than all the other treatments.

## Discussion

In this work, we prepared and characterized new nanoformulations based on polysaccharides from cyanobacteria and EPC to encapsulate natural molecules, i.e., HT and CAP (Li et al. 2020). As a first consideration, it is worth noticing that the term “nanostructure” is nowadays used in the literature to describe objects with size in different submicron ranges and here we chose to adopt this nomenclature. The results of DLS runs showed that adding lipids to particles formed by EPS only is a good strategy to obtain nanovectors with reduced size (Falsini et al. 2019; Singh et al. 2021; Falsini et al. 2023). Indeed, the mean diameter dropped from more than 900 nm to 150–200 nm. Regarding the size distribution, the system with only EPS had a PDI of around 0.9, indicating that aggregates with broad size distribution were present in solution. These particles were probably not spherical in shape, which is a typical outcome in polymer self-assembly, and the sonication used to reduce the particle size had not strong effects on these aggregates, whose mean size remained close to 1 μm.

On the other hand, the moderately negative surface charge measured for plain and loaded nanoparticles suggested that these formulations are not likely to be toxic by electrostatic interactions with the target organisms. The newly prepared samples were tested to evaluate their efficacy against *C. capitata* eggs and first instar larvae. It was observed that the system of the nanovectors themselves showed high biocompatibility, with similar larvae survival rates as the control group (Fig. 3) and had no effect on adult deterrence (Fig. 5).

Systems containing bioactive molecules at the higher concentrations exerted a greater effect of toxicity on eggs and first larvae survival. In particular, in EXP_(c),_ our nanoformulations markedly increased the efficacy of HT with respect to the pure HT exhibiting strong toxicity on the eggs. Besides, HT deters female fruit flies from landing on fruits; this deterrent effect is significantly enhanced when HT is loaded onto the nanocarrier EPS-EPC_(c)_, resulting in fewer female flies visiting the fruit. HT is one of the polyphenolic compounds, together with oleuropein, oleocanthal and lignans, found in the extract of olive mill wastewater, a widely used by-product for its antimicrobial properties (Yangui et al. 2010; Drais et al. 2021). Besides, it has been observed that filtrates from olive mill wastewater, containing polyphenols of different molecular weights ranging from 0.1 to 1 kDa, had a toxic effect on *C. capitata* (Di Ilio and Cristofaro 2021). Similar papers are present in the literature studying the effect of olive by-products containing polyphenols for the control of insects (Debo et al. 2011; Boutaj et al. 2020). Specifically, to the best of our knowledge, there are no references that report HT as a purely natural molecule applied to insect control. Thus, we proposed here innovative nanopesticides based on the loading of this natural molecule, which is able to enhance the efficacy not only on the eggs hatching but also as repellent against adult females. The possible advantage of encapsulating HT into the nanoformulations consists in multiple benefits, since a reduced amount of effective dose can be used, with consequent reduction of phytotoxicity on the treated plants if administered at high concentration (Debo et al. 2011).

Nanoformulations encapsulating CAP at a concentration of 16 mM were effective against adult females, acting as a deterrent and reducing the landing of the females on the fruits compared to the bioactive molecule alone. A dose-dependent effect against the red flour beetle (*Tribolium castaneum*) has been proved in trials where capsaicin, at the concentration of 0.1 g/mL, was microencapsulated in chitosan improving insecticidal efficiency compared to the pure molecule (Cui et al. 2023). In a recent study, Li et al. (2020) demonstrated that females displayed ovipositional avoidance of capsaicin in a dose-dependent manner. The oviposition deterrence on *Drosophila suzukii* was exerted by capsaicin at concentrations ranging from 10 to 80 mM. Our results suggest that *C. capitata* is more resistant than *D. suzuki* to capsaicin since the pure molecule at 16 mM concentration was not effective. Here, we showed the efficacy of low concentration CAP loaded in nanovectors as an insect repellent agent.

## Conclusions

In conclusion, this work showed that nanoformulates based on EPS-EPC were able to enhance the efficacy of bioactive molecules against Medfly. We think that the effect of enhancing the properties of the (biochemically labile) active molecules studied in this work is the result of more than a single advantage of administration via finely dispersed particles. Specifically, we can mention (i) increasing of bioavailability through improved affinity for water environments and amphiphilic barriers in the target systems; (ii) providing a shield from biochemical degradation and (iii) allowing sustained but efficient release, due to the extremely flexible structure of the carriers.

These findings open new perspectives for further investigations in order to find the optimal concentration and nanovector composition able to control *C. capitata* as well as other pest insects. Therefore, the EPS-EPC vector could represent a valuable tool for insect management and control with reduced harm to the environment and non-target species.

## Supplementary information

ESM 1 (XLSX 28.2 kb)

ESM 2 (XLSX 98.3 kb)

ESM 3 (XLSX 11.2 kb)

## Data Availability

The data has been made available and uploaded as article content.
